# The Impact and Effectiveness of Weight Loss on Kidney Transplant Outcomes: A Narrative Review

**DOI:** 10.3390/nu15112508

**Published:** 2023-05-28

**Authors:** Gerardo Sarno, Evelyn Frias-Toral, Florencia Ceriani, Martha Montalván, Beatriz Quintero, Rosario Suárez, Eloísa García Velasquèz, Giovanna Muscogiuri, Antonio Iannelli, Vincenzo Pilone, Luigi Schiavo

**Affiliations:** 1 San Giovanni di Dio e Ruggi D’Aragona, University Hospital, Scuola Medica Salernitana, 84131 Salerno, Italy; 2 School of Medicine, Universidad Espìritu Santo, Samborondòn 091952, Ecuador; 3 Nutrition School, Universidad de la República (UdelaR), Ricaldoni s/n, Montevideo 11300, Uruguay; 4 School of Medicine, Universidad Catòlica Santiago de Guayaquil, Av. Pdte. Carlos Julio Arosemena Tola, Guayaquil 090615, Ecuador; 5 School of Medicine, Universidad Técnica Particular de Loja, Calle París, San Cayetano Alto, Loja 110101, Ecuador; 6 Clinical Nutrition Service, Grupo Hospitalario Kennedy, Guayaquil 090615, Ecuador; 7 Endocrinology Unit, Department of Clinical Medicine and Surgery, University of Naples “Federico II”, 80131 Naples, Italy; 8 Department of Clinical Research and Innovation, University Hospital of Nice, Cimiez Hospital, 06000 Nice, France; 9 Digestive Surgery and Liver Transplantation Unit, University Hospital of Nice, Archet 2 Hospital, 06200 Nice, France; 10 Inserm, U1065, Team 8 “Hepatic Complications of Obesity and Alcohol”, 06204 Nice, France; 11 Department of Medicine, Surgery and Dentistry “Scuola Medica Salernitana”, University of Salerno, 84081 Baronissi, Italy; 12 National Biodiversiy Future Center, 90133 Palermo, Italy

**Keywords:** obesity, bariatric surgery, chronic renal insufficiency, chronic kidney failure, kidney transplantation

## Abstract

Obesity is a worldwide epidemic that leads to several non-communicable illnesses, including chronic kidney disease (CKD). Diet and lifestyle modifications have shown a limited impact in the treatment of obesity. Because the group of end-stage renal disease (ESRD) patients examined in this study had limited access to kidney transplantation (KT), patients with obesity were thought to be at an increased risk of intraoperative and postoperative KT complications. Although bariatric surgery (BS) is now recognized as the gold standard treatment for morbid obesity, its role in ESRD or kidney transplant patients remains unknown. It is critical to know the correlation between weight loss and complications before and after KT, the impact of the overall graft, and patients’ survival. Hence, this narrative review aims to present updated reports addressing when to perform surgery (before or after a KT), which surgical procedure to perform, and again, if strategies to avoid weight regain must be specific for these patients. It also analyzes the metabolic alterations produced by BS and studies its cost-effectiveness pre- and post-transplantation. Due to the better outcomes found in KT recipients, the authors consider it more convenient to perform BS before KT. However, more multicenter trials are required to provide a solid foundation for these recommendations in ERSD patients with obesity.

## 1. Introduction

Obesity, defined as excess body fat, has become a global epidemic and a major public health problem; it is estimated that by 2025, 6% of men and 9% of women will have severe obesity. It has been found that those with it have a 1.5 times greater risk of premature mortality from all causes than their normal-weight counterparts [1,2]. Obesity and its complications are known risk factors for the onset and progression of chronic kidney disease (CKD). Although body mass index (BMI) is inversely related to kidney function, it is not the only explanation for this relationship [3]. Obesity contributes to the onset of non-communicable illnesses such as arterial hypertension (AHT), diabetes mellitus (DM), and atherosclerosis, all factors that also affect the development of CKD [4]. Further, the literature indicates that in patients with CKD, weight loss reduces albuminuria and in some cases delays the decrease in the estimated glomerular filtration rate, slowing the disease’s progression [3].

Given the current global obesity epidemic, it is increasingly common for those who need a kidney transplant (KT) to have a high BMI [3]. Changes in lifestyle through healthy eating and exercise are the basis of obesity treatment under the supervision of a multidisciplinary team. In addition, bariatric surgery (BS) has been postulated to be the most effective treatment option for morbid obesity since it has been shown to lower risk factors contributing to CKD development [5,6]. It should be noted that obesity not only acts as a risk factor for the progression of this condition but is also risky in the postoperative stage and can cause complications both short- and long-term [7,8]. For its part, renal transplantation is the only curative treatment for CKD [9]. However, compared to continuing dialysis, KT appears to provide a greater survival rate for those with this condition [8]. A practical guide prepared by the Developing Education Science and Care for Renal Transplantation in European States (DESCARTES) scientific working group of the European Renal Association (ERA) suggests that BS should be considered in those patients who require a KT and who have a BMI ≥40 kg/m^2^, as well as in those with a BMI ≥35 kg/m^2^ who have at least one obesity-related condition that can improve with weight loss [8]. 

Likewise, it is essential to evaluate the most appropriate surgical technique to perform BS, depending on the case, and to contemplate its risks and benefits [10]. The time to perform BS is also a controversial aspect that must be considered, analyzing whether it is appropriate to perform it before KT, during the intervention itself, or after it [11]. 

Due to the subject’s importance, this review aims to describe the most recent evidence on BS and patients who are candidates for KT or have already received a transplant. To do this, we will inquire about bariatric interventions in KT candidates/recipients, as well as the most opportune moment to perform them. The metabolic changes caused by BS, and its cost-effectiveness in KT, will also be investigated. Similarly, dietary regimens and therapeutic strategies to avoid or minimize weight recovery will be evaluated.

## 2. Overview of Bariatric Interventions in Kidney Transplant Candidates/Recipients

### 2.1. Incidence of Morbid Obesity in This Class of Patients

Surgical scientific societies related to transplants in patients with obesity, especially kidney and liver, tend to consider bariatric interventions as a great aid to improve the possibility of these patients being on a transplant list. The Italian Society of Bariatric and Metabolic Surgery for Obesity (SICOB), for example, recently developed the first Italian guidelines for treating obesity. This group considers that for bariatric interventions in patients with BMI ≥30 kg/m^2^ with an indication for kidney or liver transplantation, bariatric or metabolic surgeries are preferable to non-bariatric or metabolic surgical treatments in order to increase the patients’ eligibility for kidney/liver transplantation, with a 95.8% agreement consensus [12]. According to research reports in different countries, the prevalence of morbid obesity in candidates for KT ranges from 6% to 30% [13,14,15,16,17]. A study in Germany found prevalence rates of obesity before and after KT of 14.8 and 19.9%, respectively, representing an increase of 34% in post-transplant individuals [13]. Another study showed a proportion of KT recipients with obesity of 9.8% (95% confidence interval, from 6.4 to 14.1%). Interestingly, this population had low adherence to the Mediterranean diet (44.7%; 40.0 vs. 45.2% in those individuals with or without obesity, correspondingly; *p* = 0.618), and 30.6% of participants had a low level of physical activity (44.0 vs. 29.1% of those with or without obesity, respectively; *p* = 0.125) [12]. In a retrospective population study including 296,807 adults (age > 17 years), KT recipients with a transplant between 2000 and 2019 presented pre-transplant BMI ≥35 kg/m^2^ results with a prevalence of 10.5% [15]. Another retrospective cohort study, with 736 adult KT recipients, aimed to assess the risk of surgical complications and found a prevalence of 14.2% with BMI ≥35 kg/m^2^ [16]. Interestingly, a 2017 meta-analysis of 165 studies (*n* = 1,534,845 participants) concluded that obesity might be a protective factor for all-cause mortality in the predialysis and hemodialysis populations; an increased risk was seen in transplant recipients at morbid obesity levels. (>35 kg/m^2^). Ladhani et al. reported that the prevalence of obesity in the US is >30% among people on dialysis, as indicated previously by Friedman [17,18]. 

### 2.2. Type of Bariatric Interventions Performed

Bariatric procedures implemented in morbidly obese patients with ESRD include Roux-en-Y gastric bypass (RYGB) and sleeve gastrectomy (SG) [10]. However, lifestyle modifications are generally unsuccessful in significant weight loss in patients with morbid obesity [19]. 

SG has several advantages over RYGB, as it is technically less challenging and faster, has a lower incidence of surgical complications, and is commonly used in candidates for KT with some degree of obesity in many transplant hospitals [9,20]. However, a decision analytic-model created by Choudhury et al. to simulate the life of patients with morbid obesity and ESRD who were deemed ineligible because having a pre-intervention BMI ≥45 kg/m^2^, revealed that when compared to SG and medical weight management, RYGB enhances opportunities for KT, consequently expands long-term survival, and improves access to KT for morbidly obese patients [10]. 

SG is commonly performed laparoscopically (LSG), which has been reported to be safe and efficient [21,22], but more recently, with the advent of new robotic technology, robotic SG is used among BS methods for some surgical specialties, such as transplants in the morbidly obese. A randomized clinical trial by Spaggiari et al. at the University of Illinois, US, compared the safety and efficacy of combining robotic SG and robotic-assisted KT (KT + SG) to robotic KT alone (KT) in candidates with class II or III obesity, reporting that graft failure rates after 1 year of follow-up were not different between the groups. However, the change in BMI was –8.76 ± 1.82 kg/m^2^ in the (KT + SG) group compared to 1.70 ± 2.30 kg/m^2^ in the (KT) group (*p* = 0.0041), with no incidence of surgical complications [19]. 

### 2.3. Is a Specific BMI Cut-Off Point in Patients That Makes Bariatric Surgery More Effective?

Before considering BS there is a critical issue of the BMI cut-off point for allowing access to KT waiting lists for high-risk ESRD candidates with obesity; this cut-off point is often between 35 and 45 kg/m^2^ [9]. Despite this, to date there is no standardized specific point. 

However, a machine learning approach used to analyze the results and characteristics of morbidly obese KT recipients concluded that obesity itself should be reevaluated as an impediment to KT because they found no differences between diverse clusters to one-year graft survival as well as patient survival in this cohort, applying unsupervised machine learning [23].

**Figure 1:**
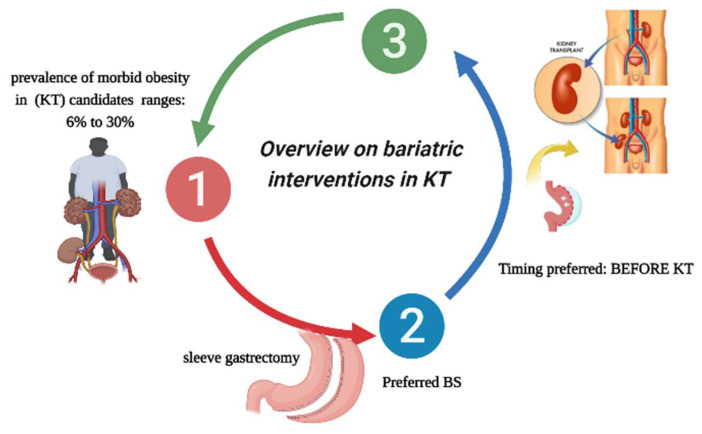
Overview of bariatric interventions in kidney transplantation (KT): (1) Research shows the prevalence of morbid obesity among KT candidates ranges from 6 to 30%; (2) The preferred BS procedure is SG; and (3) Performing BS before KT seems to be the most convenient time. BS: Bariatric surgery, KT: Kidney transplantation, SG: sleeve gastrectomy.

Despite all the advances in this field, a multicentric national research in Germany in 2022 reported that BS is only sometimes considered in every KT center in that country [9]. In the study, there was a consensus that there is no single treatment option for obese KT candidates but that an integrated approach of nutritional counseling, activity programs, and BS seems appropriate. Nevertheless, the accessibility of treatment choices at German facilities was significantly higher than that found in a Canadian study in which a survey was sent to most transplant nephrologists and surgeons across the country. This study reported that BS experience was small, most transplant centers did not have a weight management program, and that the BMI limit was a barrier to accessing the KT waiting list in Canada [24]. However, this can be explained by considering that it is uncommon in German transplant centers to handle more than 25 patients with BS prior to KT [9]. An evaluation of dietary regimes and more traditional strategies for weight loss before BS shows evidence of only a limited benefit in achieving the goal of losing weight. According to a small-sample retrospective study a traditional weight loss plan showed limited but not insignificant positive results. The authors found that 26.3% of patients had successful weight loss (BMI <35 kg/m^2^); however, no patient with a BMI >40 kg/m^2^ was successful [25]. Another study that compared KT candidates with a mean BMI of 44.4 ± 4.6 kg/m^2^ who received a traditional weight loss approach (multidisciplinary consultation) with a BS intervention showed that the traditional approach was ineffective, while BS was strongly associated with subsequent KT (HR = 8.39 [95% CI 1.71 to 41.19]; *p* = 0.009) [25]. Sarno et al. recently reported that when comparing different types of diet, such as preoperative low-calorie diet (LCD), very-low-calorie diet (VLCD), low-fat diet, intermittent fasting, or a Mediterranean diet in relation to BS complications, it seems that LCD has shown better tolerance and adherence than VLCD. Nonetheless, the authors suggest that better controlled research is still needed to define the optimum diet plan for weight loss before BS, since this issue has controversial positions [26]. It is also relevant to remember that complex patients with comorbid obesity conditions should be supervised with combined strategies before BS, including weight loss and the consumption of omega-3 fatty acids, which have been shown to reduce liver steatosis and systemic inflammation, thus improving postoperative outcomes [27]. In summary, as an overview of bariatric interventions in KT candidates/recipients, it can be concluded that recent research shows that the prevalence of obesity among KT candidates/recipients is around 30% in the US. However, reports from other countries state that it is less than 15%. Another issue to consider is that according to new publications, the most convenient surgical technology for BS is SG, considering that robotic technology could improve its results. Finally, performing BS before KT seems to be the most convenient time in terms of overall benefits for patients. Nonetheless, some studies suggest that this claim is not valid in all cases (Figure 1).

## 3. Metabolic Changes Related to Bariatric Surgery

### 3.1. Impact of Bariatric Surgery on the Absorption and Effectiveness of Immunosuppressive Drugs

Among the potential risks of BS in KT recipients are the possible pharmacokinetic alterations of immunosuppressive drugs and undesirable graft rejection. In this sense, some publications have shown that BS, depending on the type, indeed produces pharmacokinetic changes in immunosuppressive drugs, but the evidence does not show a negative clinical impact on immunosuppressive treatment or an increase in graft rejection. For example, in two studies, among kidney, liver, pancreas, or kidney-pancreas transplant recipients SG did not negatively influence the amount of immunosuppressive medication up to 1 year after the transplant [28,29]. 

In line with this, tacrolimus blood levels decreased slightly with BS among solid-organ transplant recipients but remained within the therapeutic range. Therefore, the authors suggested that LSG and laparoscopic RYGB (LRYGB) allowed good immunosuppressive management without graft rejection problems [30]. Similarly, in 11 KT recipients undergoing BS, blood concentrations remained relatively stable for calcineurin inhibitors but showed an irregular pattern for cyclosporine A. However, this did not affect the graft rejection rate in these patients 6 months after surgery [31]. 

More recently, a study reviewed the pharmacokinetics of immunosuppressive drugs (tacrolimus, extended-release tacrolimus, mycophenolate mofetil, and enteric-coated mycophenolate sodium) in ESRD patients undergoing LSG. BS altered the pharmacokinetics of all the drugs evaluated, with an increase in exposure and AUC24. For some drugs, the maximum concentrations increased while the total apparent clearance decreased. Therefore, the authors recommended increasing treatment follow-up, especially given the possible need to reduce immunosuppressive medication in patients with BS [32]. 

### 3.2. Outcomes and Complications of Transplant Surgery. Impact of Obesity on KT Outcomes

A wide variety of retrospective and cohort studies have documented increased transplant rejection and other complications among obese KT recipients. In general, compared to normal weight/overweight recipients, the following outcomes have been documented among obese patients: higher incidence of death, cardiac problems, surgical complications, wounds or other bacterial infections, fascial dehiscence, and lymphoceles [33,34]. Other complications observed more frequently among obese subjects were primary renal failure, delayed graft function, a high incidence of loss of graft function, and even significantly lower glomerular filtration rates 1 year post-transplantation [35]. In addition, lower patient and graft survival has been described, after 1, 5, and up to 10 years of follow-up in obese versus non-obese recipients [36]. In line with this, a meta-analysis of 17 observational articles that included more than 130,000 patients found an association between recipient obesity and a higher incidence of graft rejection and death associated with graft rejection [37]. However, some studies have not found a higher incidence of complications in obese versus non-obese KT recipients. For instance, the prevalence of delayed graft function, reinterventions, readmissions, wound-related complications, patient survival, or kidney function at 1 year did not differ significantly across the groups as reported by a more recent single-center retrospective study that compared BMI ≥40 patients (n = 84, BMI = 42 ± 2 kg/m^2^) to a matched BMI < 40 cohort (n = 84, BMI = 28 ± 5 kg/m^2^) [38].

The degree of recipient weight loss influences the results after transplantation. Illustrating this, a cohort study that evaluated the results of KT in 7270 patients found more significant graft survival in non-obese patients compared to their counterparts. It is noteworthy that a higher graft survival was also found in patients with obesity who lost more than 10% weight compared to patients with obesity and without weight loss. However, that research did not find a relationship between obesity or weight loss with mortality after transplantation [39]. 

It is well known that the weight of the organ donor can influence transplant outcomes for the recipient. A cohort study of more than 200,000 KT recipients compared donor–recipient pairs based on the prevalence of obesity. Graft rejection was associated with recipient obesity, and the risk of rejection was higher when both donor and recipient were obese [40]. 

Despite the evidence, the complete role of recipient obesity as a risk factor for poor outcomes after KT remains unclear. Among the investigations that failed to find a relationship between obesity and poor outcomes is a cohort study of more than 17,000 renal recipients with BMIs from <18.5 to ≥40 kg/m^2^ followed for more than 5 years. The authors found no differences in patient survival or graft survival between patients with different BMIs [41]. More recently, a meta-analysis of 17 prospective and retrospective observational articles found no relationship between recipient BMI and the incidence of early hospital readmission within 30 days after KT [42]. 

### 3.3. Impact of BS on Weight Loss and Outcomes in KT Patients

Considering the evidence, it is strongly recommended to encourage patients with obesity to lose weight and maintain an adequate weight before KT [8]. Therefore, it is essential to establish successful treatments to lower the risk of poor outcomes in patients undergoing interventions to reduce obesity, mainly BS. 

To date, controversial results related to the optimal time to perform BS have been reported, although most researchers recommend a “before KT” approach. Here are some scientific findings that illustrate the controversy on this point. First, some studies show greater benefits of BS performed before KT. A recent systematic review of 31 articles published in 2022 reported that 18 studies found a 13.7% graft loss and 9.1% mortality within 5 years post-transplantation in patients without pretransplantation BS. Nevertheless, among recipients with pretransplantation BS, 15 studies showed 8.7% graft loss and 2.8% mortality between 1 month and 5 years after transplantation [2], suggesting a better performance with BS before KT. Similar findings were reported by Schindell et al. in a retrospective case-control study with a mean follow-up of 2.4 ± 1.3 years. In this research, the authors described a significant decrease in glomerular filtration rates and lower comorbidities (*p* < 0.001) in patients who underwent BS before KT compared with the controls [11].

On the contrary, some studies show no difference in weight loss or outcomes in patients with BS before vs. after KT. A study by Cohen et al. found equivalent results in maintaining weight loss and improving long-term survival of allograft with BS before and after KT compared to matched controls, suggesting that BS is a safe and reasonable approach to weight loss before and after transplantation [20]. As in other transplanted organs, the tendency is to perform BS before the transplant for better results, such as for liver transplantation [43].

In this regard, a retrospective study examining patients undergoing BS before (n = 43) or after (n = 21) KT found increased graft survival in BS patients versus controls. However, they found no difference in weight loss between patients who underwent BS before or after KT [44]. Similarly, a prospective clinical trial following eight KT candidates with obesity (mean BMI 38.8 kg/m^2^) and undergoing LSG showed beneficial effects of BS. Approximately 3 months after BS, the BMI dropped below 35 kg/m^2^, and one year after BS, 62.7% of body mass was lost. KT was performed approximately 17 months after successful BS, resulting in good graft function for up to 3.2 ± 1.4 years of follow-up [21].

At this point, a thorough dissection of the available literature reveals the need for more and better-conducted studies to assess the true impact of BS on KT outcomes. To illustrate this issue, we examine a systematic review by Mousapour et al., who aimed to understand the impact of BS on KT outcomes. They reviewed 31 articles with more than 2000 KT candidates with obesity. The authors found no differences concerning graft loss, patient mortality, delayed graft function, wound complications, and lymphocele between patients who received BS and those without BS after a follow-up time of between 1 month and more than 5 years. We consider that very marked differences between the groups of articles contrasted did not allow them to establish accurate comparisons. For example, the methodological quality of the studies, the proportion of living donors, and the total number of subjects evaluated were much lower in the investigations related to patients with obesity that underwent BS. The type of BS and the follow-up period were described in only some cases. In addition, there were studies with differing subject follow-up periods [2]. 

### 3.4. Diet Regimens and Therapeutic Strategies to Avoid or Minimize Weight Regain

Weight regain has been described in some studies, which report a high frequency of patients who gained weight after KT. Cohort studies in Brazil and Saudi Arabia determined that 72.7% and 54.6% of patients gained weight after 1 year of KT, respectively [45,46]. 

It is important to consider that obesity/weight gain after KT is multifactorial. Some receptor factors related to weight gain are older age [13], female gender [13,45,47], lower hospitalization rate [45], creatinine level [47], and lower body weight or obesity before KT [45,46]. Having received kidneys from a living or younger donor was also related to weight gain [45,46,47]. Conversely, some studies have found no relationship between using steroids or calcineurin inhibitors with weight gain or increased anthropometric measurements in KT recipients [47,48]. 

Notably, the possibility of gaining weight after the KT could differ depending on the patient’s initial weight before the KT. For example, a retrospective study in 131 patients classified as malnourished, eutrophic, or with obesity found a highly significant weight gain 6 and 12 months after KT in all groups of patients. The problem is that although a good proportion of previously malnourished and eutrophic patients gained weight, none became obese 12 months after KT. In contrast, among patients who were initially overweight or obese, 28.6% and 100% were obese at 6 months post-RT, respectively [49]. Diet regimens are aids in weight loss when used before or after BS. For example, administering a VLC-ketogenic diet for 30 days prior to BS is safe and effective for weight and visceral fat reduction, blood glucose lowering, and improving the lipid profile [50]. 

As explained in detail by Schiavo et al., 2017, in people with obesity there is a more significant amount of fat mass (FM) and fat-free mass (FFM) compared to that found in women and men of normal weight [51]. Considering the importance of maintaining FFM as a metabolically active mass, it is recommended that weight reduction measures be applied to achieve a reduction in FF and FFM until the levels reach what patients would have under normal weight conditions. Emphasis is also placed on measuring body composition before and after BS to ensure the quantification of changes in FM and FFM [51]. On the other hand, in patients with BS, an adjustment of the amount of protein and the general composition of the diet is suggested [51]. Providing enriched/high protein diets to individuals with obesity before and after LSG has produced beneficial effects. For example, a Mediterranean protein-enriched diet administered to subjects with obesity for 8 weeks before BS reduced weight, visceral fat, liver size, and fat mass, with the advantage that it did not produce a significant loss of FM [52]. Similarly, a high protein diet administered postoperatively up to 12 months after BS reduced FM more and FFM less than a normal protein diet [53]. 

### 3.5. Nutritional Behavior

Guiding and monitoring diet and eating habits after BS is fundamental to maintaining weight loss and avoiding nutritional deficits and metabolic disorders. To illustrate this point, we analyzed the results of a 4-year follow-up study of patients undergoing SG, in which information on their dietary habits was collected and compared with the recommendations of the Italian Mediterranean diet. Among the positive results, the improvement and/or remission of obstructive sleep apnea (100%), type 2 diabetes (73%), and to a lesser extent, hypertension (64.7%) stand out. Unfortunately, the authors noted several unhelpful findings. First, they found an inadequate intake of fruit, vegetables, poultry, and complex carbohydrates, as well as anemia and deficiencies in vitamin B12, vitamin D, folic acid, and iron. All this is combined with a deficient consumption of micronutrient supplements. Second, weight recovery occurred in 37.8% of the participants. It must be taken into account that one in three subjects who gained weight had these three unfavorable habits: low consumption of vegetables and fruits; high consumption of rice, pasta, bread, and potatoes; and no physical activity [53]. 

Finally, nutritional behavior was evaluated after a follow-up period of 94 ± 67 months after KT in 154 patients with a BMI of between 19.1 and 40.8 kg/m^2^ (45.5% overweight and 16.2% obese). Unfortunately, food consumption was not balanced. The daily amount consumed per patient was very high for meat, bread, potatoes, sweet foods, and salt. The patients ingested excessive food with a high energy density, especially with large amounts of saturated fatty acids. The authors speculate that the rigorous dietary habits followed by these patients while they had kidney disease were lost after KT [54].

Given these findings, it is highly desirable that KT candidates and recipients are closely guided and monitored concerning diet and nutritional status, as recommended elsewhere [8]. 

In KT recipients, BS can produce pharmacokinetic changes in immunosuppressive drugs, which do not appear to have a negative clinical impact on immunosuppressive therapy or increased graft rejection. However, close monitoring is recommended due to the possible need to adjust immunosuppressive medication in patients with BS. Analyzing the evidence, it is strongly encouraged that patients with obesity lose weight and maintain an adequate weight before and after the KT; this is where BS is crucial. BS is effective in reducing weight in KT candidates and recipients, but there is a need for more and better studies to assess the true impact of BS on KT outcomes. Guiding and controlling diet and eating habits after BS is fundamental to maintaining weight loss and avoiding nutritional deficits and metabolic disorders. 

## 4. Cost-Effectiveness of Bariatric Surgery in Kidney Transplantation

Obesity is rising among the adult population, even in patients with kidney failure who need transplants. Obesity is also linked to inferior transplant outcomes and is a relative contraindication to organ transplantation [42]. KT is a life-improvement treatment that results in lower hospitalization rates and significant long-term cost savings to health systems compared to dialysis. The expected health costs over 10 years were avoided through KT, resulting in a cost savings of EUR 380,000 per patient compared with no transplantation, independent of patient age [55]. 

Some reports mention that BS may be used as a bridge for patients in need of transplantation who are not eligible for it because of their morbid obesity [56]. There is still no guideline or consensus on the highest BMI to be considered a contraindication to KT. Most guidelines strongly suggest that weight loss must be encouraged for patients with BMI >30 kg/m^2^ [57]. 

The effect of the degree of obesity studied on KT patients describes an increased patient survival and graft survival following KT when comparing patients with a BMI of 30–35 and those with a BMI >35 at 1 and 5 years after transplantation [56]. Many large studies have shown that obesity among KT recipients is linked with a higher risk of complications after the procedure, such as allograft failure and death. Patients with a BMI of 34–36 kg/m^2^ had a higher risk of post-transplant death, indicating that KT may carry an unacceptable level of risk in these patients and that the benefit of transplant should be weighed against the risk of continuing on dialysis [58]. 

BS may benefit select end-stage organ disease patients with obesity [57], increase the transplant candidacy of patients with obesity and ESRD, and potentially improve immediate and late outcomes. Obesity comorbidities are reported as the leading cause of ESRD. Patients with obesity on waiting lists can develop several comorbid conditions requiring temporary wait-list suspension. The higher mortality risk seen in patients with obesity during the peritransplant period may be connected with concurrent comorbid disorders that could deteriorate in the post-transplantation period or be the outcome of peritransplant complications. The most commonly performed BS procedures in patients with ESRD and transplant patients are LSG and LRYGB [59]. 

Weight loss in patients with ESRD is extremely difficult due to many reasons, such as restrictions on a renal diet, limited exercise tolerance due to coexisting comorbid conditions, and dialysis-related fatigue. Medical management could have limited long-term success, but it takes a longer time, while BS could offer a reliable strategy to achieve weight loss in KT candidates [59]. 

Yemini et al. concluded that LSG and LRYGB appear to effectively work in patients with obesity because they address obesity issues before KT and improve surgical access for them to the waiting list for KT. Morbidly obese KT candidates would benefit from prior BS. It is critical to mention that all patients underwent a multidisciplinary evaluation by a dietitian, psychologist, anesthesiologist, bariatric surgeon, and other specialist consultants as needed prior to the BS [57]. LSG may be preferred in the ESRD population and may provide considerable benefits over LRYGB, such as a simpler and quicker surgical process and a reduced risk of complications related to surgery [59].

Many risks should be considered prior to a BS. Complications from surgical interventions in kidney recipients may be higher than in the general population. Furthermore, BS can impair the absorption of immunosuppressive drugs [60]. This concern would highlight the importance of closely monitoring immunosuppressive concentrations following BS, mainly during the first few months following the procedure [59].

BS is linked to significant weight loss (from 29.8% to 72.8%) in ESRD patients, with documented death and morbidity rates of 2% and 7%, correspondingly [59]. Some benefits from BS are obesity-related comorbidities remission, defined as a complete withdrawal of medical treatment with normal laboratory results or improvement of medication dosage. Some improved comorbidities are type 2 diabetes, dyslipidemia, and a decrease in patients’ cardiometabolic risk [57]. These positive outcomes mean that the benefits of weight loss they achieved may help improve survival while waiting for a suitable organ donor [42]. 

As expected, ESRD patients experience more post-bariatric surgical problems than non-ESRD patients. The death rate reported in various BS cohorts (0.18% in meta-analysis) can be 10 times higher than the mortality risk in patients with ESRD and no BS evaluated by this meta-analysis (2%; 95% CI: 0–3%) [61]. 

Another study showed that BS has significant and sustainable effects on weight loss, can improve transplant candidacy effectively, and can successfully move patients through the care pathway to transplantation. Since around 14% of patients with CKD who receive hemodialysis therapy suffer from severe obesity, increasing their chance of transplant eligibility could also result in significant long-term cost savings to health systems [42]. 

In addition, when evaluating a patient candidate for BS, the transplant specialist must weigh the risks of the procedure in class II–III obese patients with chronic renal failure, taking into account the potentially increased success rate of KT in a recipient who is less obese versus the combined risks of KT in an immunosuppressed patient with diabetes. There is currently conflicting evidence on the viability and safety of BS for certain people [57]. Following transplantation, weight loss brought on by BS seems to have a good effect on how well the grafted kidney functioned; however, immunosuppressant medication had to be carefully monitored [61].

Few studies have analyzed the role of BS after KT in morbidly obese recipients. BS in KT recipients may be associated with an increased operative time, length of stay, readmission, and surgical site infection but not with increased mortality. Following kidney transplantation, BS is linked to considerable and persistent weight loss, a decrease in comorbidities, and an enhancement in graft function without significantly changing immunosuppressive treatment absorption [59].

**Figure 2:**
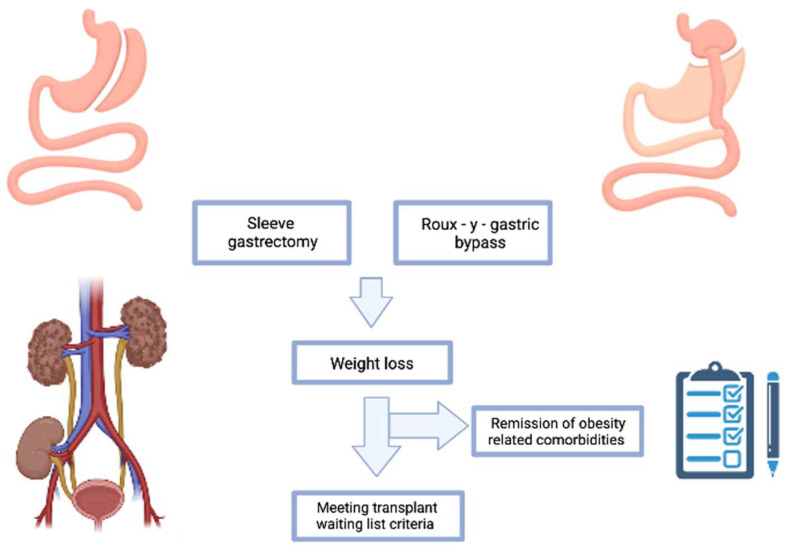
Cost-Effectiveness of Bariatric Surgery in kidney transplantation. Bariatric surgery can be done before kidney transplantation. The main goal is to meet the proper weight-listing requirements and reduce the risk of perioperative-transplant morbidity and mortality. After the bariatric surgery there is a reduction of comorbidities related to obesity.

There is currently no recommendation for the best kind of BS to perform in patients awaiting transplantation or who have already received one. Additional research should compare patients who had BS with those who did not have it regarding access to KT. It is essential to discuss the prognosis of people who have BS but are still receiving dialysis (Figure 2) [61].

## 5. Future Perspectives

KT can offer better survival rates for patients with and without obesity, so meeting the list requirements and reducing the patients’ comorbidities is vital. The best type of BS and the time to perform it before KT remains to be elucidated [59]. Chronic kidney failure (CKF) is made worse by obesity. Patients with severe obesity stages, defined as classes II and III, with a BMI ≥35 kg/m^2^ are candidates for BS, which is the most effective way to lose weight for a more extended period of time. 

Weight loss may alleviate or reverse diabetes and hypertension, improve adipocytokine profiles, and withdraw glomerular hyperfiltration and albuminuria, among other favorable outcomes [62]. Obesity-related cardiometabolic comorbidities such as hypertension and type 2 diabetes also worsen kidney function with an increased risk of glomerulosclerosis and nephropathy, so weight loss is a defining factor. The mean percentage of body weight loss for individuals with obesity using nonsurgical methods is from 5 to 10% of their initial weight. In patients with class II (BMI ≥ 35) or class III obesity (BMI ≥ 40), such a small reduction is often insufficient to improve their access to be eligible for KT. BS might allow them to lose enough weight quickly, permitting them to improve renal function and comorbidities. The metabolic benefit would likely be significant for this group of patients [61]. 

Pretransplant BS is innocuous and may make transplantation more accessible to individuals with obesity. There are two types of possible surgery, LSG and LRYGB, and both appear to work effectively in patients with obesity because they address obesity problems before KT and improve surgical access for them to the list of KT [59]. 

LSG could be preferable and offer significant advantages over RYGB, including a more effortless and faster surgical procedure and a lower incidence of surgical complications. In pretransplant, LSG results in sustained weight loss and is associated with improving obesity-related comorbidities. Moreover, LSG appears not to alter immunosuppressive pharmacokinetics, avoiding under- and over-immunosuppression [59]. Alterations in pharmacokinetics with the immunosuppressants have been observed in patients who underwent RYGB related to the characteristics of this procedure, which is associated with gastric restriction and a degree of malabsorption [44]. 

In comparison to the matched controls, BS leads in equivalent maintenance of weight loss and enhanced long-term allograft survival. In order to lose weight both before and after transplantation, BS seems to be an effective and proper option [44]. 

**Figure 3:**
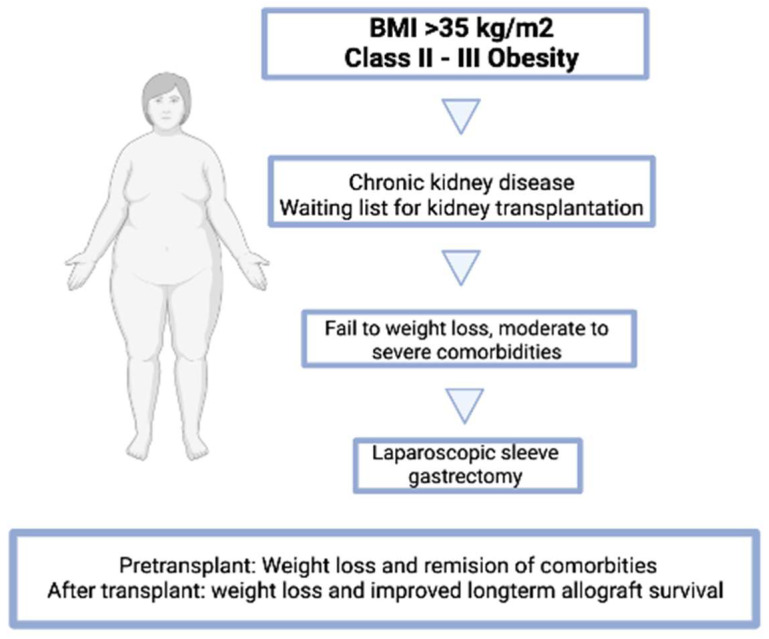
Chronic kidney disease, obesity class II–III, and bariatric surgery. Patients with chronic kidney disease and waiting for transplantation can have bariatric surgery with the benefits of weight loss and remission of their comorbidities while on the waiting list. Bariatric surgery after transplant can improve long-term allograft survival. LSG is preferable for its fewer complications. BMI = body mass index.

Any attempt should be made to improve the results for these patients, and obesity should not rule out the prospect of a KT. It has been established that BS can reduce obesity- and weight-related comorbidities while improving access to KT waiting lists and patient safety. Future research should assess the possible contribution of BS to the management of post-transplant problems in recipients with obesity and the long-term decrease of cardiovascular problems (Figure 3) [59].

## 6. Conclusions

Obesity is a serious public health problem, and its presence and associated comorbidities impact the development and evolution of CKD. Based on the need to perform a KT, different scientific societies recommend losing body weight to improve the procedure’s short- and long-term outcomes. Changes in diet and lifestyle are essential but often insufficient, so BS is considered an aid. This will allow control of associated comorbidities as well as increase the chances of KT candidacy.

RYGB and SG are among the different surgical techniques to reduce weight. The latter consists of a simpler intervention with a lower risk of complications; however, RYGB has been shown to increase long-term survival compared to SG. 

Another critical aspect to consider is the most appropriate time for BS. Many publications recommend performing it prior to KT since it reduces the possibility of graft loss and post-transplant mortality. However, other studies have shown benefits even if the intervention is performed after KT. 

Although it is common for the BMI cut-off point for KT to be between 35 and 45 kg/m^2^, there is no consensus on this. Although BS is one of the options, more traditional strategies are also used to achieve the desired weight loss. Despite the lack of consensus on the ideal diet to follow, the LCD has been shown to have the most benefits, since patients tolerate it well. 

The risk of immunosuppressive drug pharmacokinetic changes and undesirable graft rejection is a critical factor to consider in patients with BS. Some drugs have been found to decrease slightly in effectiveness with BS but remain within the therapeutic range. Other studies suggest that in patients undergoing LSG, for some drugs, the maximum concentrations increased while the total apparent clearance decreased; therefore, frequent patient monitoring during this period is essential to make the necessary adjustments to the medication. 

Undoubtedly patients with obesity who must receive a KT will benefit from losing weight, but work must be done so that after having lost weight, an increase does not occur again. Studies have revealed that a considerable percentage of individuals gain weight after KT. Following the intervention, it is critical to teach the patient healthy eating habits in order to maintain weight loss and avoid nutritional deficiencies.

## Data Availability

The data is available through a specific request to the authors.
